# A method to control terpineol production from turpentine by acid catalysts mixing

**DOI:** 10.1016/j.heliyon.2020.e04984

**Published:** 2020-10-08

**Authors:** Tirto Prakoso, Ilham Ardiyanto Putra, Lienda Handojo, Tatang Hernas Soerawidjaja, Haryo Pandu Winoto, Antonius Indarto

**Affiliations:** a Department of Chemical Engineering, Institut Teknologi Bandung, Bandung, Indonesia; b Department of Bioenergy Engineering and Chemurgy, Institut Teknologi Bandung, Sumedang, Indonesia

**Keywords:** Chemical engineering, Natural product chemistry, Catalyst, Chemical reaction engineering, Industrial chemistry, Chemical synthesis, Turpentine, Terpineol, Hydration reaction, Acid catalysts

## Abstract

Terpineol, a promising valorisation product of pine industry, is widely used as an active ingredient for disinfectant soap, cleansers, perfumes, and pharmaceutical purposes. Synthesis of terpineol is generally carried out by separation of α-pinene compounds from crude turpentine through fractionation and then hydrated (addition of water) with the help of acid catalysts. However, direct turpentine hydration without pre-fractionation process can be more beneficial from economic and process point of views. This study aims to investigate the effect of both single and mixed/combined catalysts towards terpineol yield. Combined strong and weak acid catalysts were required to obtain high feed conversion and terpineol yield. The selectivity of terpineol is then correlated to the solubility of a weak/organic acid. In this study, the highest yield of terpineol was 54.0 ± 8.2%-w/w using combination of formic acid and sulphuric acid.

## 1 Introduction

Pine (*Pinus merkusii*) is one of the biggest natural sources of turpentine oil production. In daily application, turpentine oil (a mixture of various monoterpene hydrocarbons) is used as a paint, varnish, coating, and an organic solvent. Turpentine was obtained from the separation of pine gum as a light product (distillate) of the fractionation process. Although it could be sold directly, several turpentine derivatives, such as geraniol, menthol, terpineol, and cineol, have higher market price [1, 2, 3]. The worldwide demand for pine derived chemicals including turpentine and its derivatives are projected to reach 5.27 billion USD by 2021 [4] with Indonesia as the third largest producer after China and Brazil. One important derivative of turpentine is terpineol that mainly used as disinfectant for soap, flavouring agent, and pharmaceutical substances [5, 6].

In order to produce terpineol, raw turpentine has to be fractionated first to produce α-pinene followed by hydration under acidic condition [7, 8]. Until now, this pathway is believed as the best route to produce terpineol in order to minimize the side products of the reaction into β-pinene, limonene, or δ-carene. However, the fractionation process requires lengthy operating time and high energy consumption (for heating/cooling and vacuum generation). Elimination of the fractionation step by directly hydrating raw turpentine into terpineol can possibly lead to a more economical process. Commercial terpineol production from turpentine itself consists of two reaction steps as following: (1) hydration of turpentine into terpin hydrate (C_10_H_22_O_3_) and (2) selective dehydration of terpin hydrate into terpineol [9, 10].

Despite its proven effectivity, the aforementioned pathway is less preferred because it produces terpin hydrate as intermediate solid, requires long reaction time (*ca*. 20 h), and uses toluene as its solvent [11, 12]. Another promising alternative is to conduct the reaction through one-pot reaction in which turpentine is directly hydrated into terpineol. This alternative can be carried out at high reaction temperature and catalysed by acid catalyst with shorter reaction/mixing times. Unfortunately, compared to two-steps pathway that has up to 75% yield, the second alternative produces less terpineol. Thus the remaining challenge of the aforementioned is finding a suitable acid catalyst to drive the hydration reaction into terpineol.

Conversion of turpentine (especially α-pinene component) into terpineol can be carried out by hydrating the double bond functional group within pinene structure (addition of one water molecule). This reaction begins with the transformation of one double-bond into single-bond of C–C to form carbocation with the help of an acid catalyst [13]. There are five carbocation species, pinanyl, p-menthenyl, isobornyl, fenchyl, and terpinene, have been identified with relatively different in term of reaction activation energy [14]. These carbocations will be attacked by water molecules (as a nucleophile) forming a hydroxyl bond and become terpineol. However, this reaction tends to form by-products such as fenchol, borneol, cineol, and others. By-products formation has been reported as the effect of isomerization reaction via terpinene carbocation formation [14]. Previous works have been conducted in order to synthesise pure α-pinene to terpineol focusing on the acid catalyst selection [14]. Studies on the type of acid catalysts were done by applying: (1) sulfuric acid and resulting 47% of terpineol yield [10]; (2) Heteropolyacid and producing 90% of α-pinene conversion with 30% of terpineol selectivity [15]; (3) mixed hydrochloric acid, acetic acid, oxalic acid and chloroacetic acid and resulting 91.2% of α-pinene conversion with 49.2% of terpineol selectivity [3]. Utami's group attempted to convert raw turpentine into terpineol by using direct method and obtained terpineol selectivity of 54% using chloroacetic acid [16]. Furthermore, based on the previous research [16], the optimal conditions of α-pinene reaction with chloroacetic acid was achieved at a temperature of *ca*. 80 °C. The optimum reaction time and the amount of water addition in α-pinene reaction were also studied [17] and conclusively they found the optimum time reaction was 6 h with water to α-pinene ratio of 10 mol/mol. Subsequent work by [18] on turpentine synthesis shows that the highest yield of terpineol was obtained at 85 °C of reaction temperature.

Based on the previous studies, crude turpentine feed produced lower terpineol yield (generally below 40%) compared to α-pinene one [18]. Therefore, the objective of this study is to maximize the yield of terpineol by selecting acid catalyst(s) or combination of them and optimizing the reaction condition. Since the results of this study will be applied for larger production, the proposed acid catalysts should be cheap and abundant in the market.

## 2 Experimental setup

Raw turpentine was kindly supplied from Perhutani Pine Chemical Industry (PPCI), Pemalang, Indonesia and used without any further treatment. The raw feed consisted of 79.1% of α-pinene and the rest were other isomers, such as β-pinene (2.9%), δ-carene (13.3%), and δ-limonene (1.1%). There were two methods of acid-catalyst(s) utilization in this work (complete experimental sequence and set up is explained in detail in Supplementary Material-Figure S1). Those aforementioned methods are (1) utilization of single/individual catalyst (only weak/organic acid) and (2) mixed/combined catalyst of weak/organic acid and a strong/inorganic acid. Briefly, the mixture of turpentine oil, demineralized water, and acid catalyst(s) was heated and stirred in the reactor at varied temperatures. The heated reactor was equipped with a reflux condenser at top of the system to ensure that no vapour left the system. The reaction resulted in two-layers liquid formation and the separation was conducted using a separation funnel. The top layer was ‘oil layer’ composed of residual turpentine, terpineol, and by-products while the bottom layer was ‘water layer’ composed of acid and water residues [19]. After the phase-separation and adsorption for impurities removal, the product was analysed by Gas Chromatograph-Mass Spectrometry (Shimadzu GCMS-QP-2010 with Rtx-5MS capillary column) at conditions of 200 °C injection temperature, 70–190 °C column temperature with initial temperature of 70 °C for 2 min and heated up to 190 °C and held for 6 min with ramping of 30 °C/min. The GCMS was useful to identified all possible reaction products [20, 21, 22, 23] and able to separate clearly all chemical products clearly. The calibrated curve are shown in Supplementary Material-Figure S2.

In this study, the type and mixing ratio among the catalysts (strong inorganic and weak organic acids) were the main investigated parameters. The strong acid catalysts (phosphoric acid, p-toluenesulfonic acid (PTSA), and sulfuric acid) were introduced to increase the conversion of the feed while weak acids (oxalic acid, citric acid, and formic acid) used to direct the selective dehydration reaction of terpin hydrate into terpineol. Complete experimental acid catalyst variation is tabulated in Table 1.

**Table 1:** Acid catalyst variation experiments.

Catalyst variation	Mol Ratio of Variation	Temperature (^o^C)	Reaction Time (h)
Experiment A: Optimum Reaction Condition			
- PTSA/Water/Turpentine	1/5/1; 1/10/1; 1/15/1	75; 85; 95	4; 6; 8
Experiment B: Single catalyst of weak acid			
- Oxalic Acid/Turpentine	0.5/1; 1/1; 1.5/1; 2/1	85	6
- Citric Acid/Turpentine	0.5/1; 1/1; 1.5/1; 2/1	85	6
- Formic Acid/Turpentine	0.5/1; 1/1; 1.5/1; 2/1	85	6
Experiment C: Combined strong and weak acid catalyst			
- Phosphoric Acid - Oxalic Acid - Turpentine	0.2/1/1; 0.05/1/1	85	6
- PTSA/Oxalic Acid/Turpentine	0.2/1/1; 0.05/1/1	85	6
- Sulphuric Acid/Oxalic Acid/Turpentine	0.2/1/1; 0.05/1/1	85	6
- Phosphoric Acid/Citric Acid/Turpentine	0.2/1.5/1; 0.05/1.5/1	85	6
- PTSA/Citric Acid/Turpentine	0.2/1.5/1; 0.05/1.5/1	85	6
- Sulphuric Acid/Citric Acid/Turpentine	0.2/1.5/1; 0.05/1.5/1	85	6
- Phosphoric Acid/Formic Acid/Turpentine	0.2/2/1; 0.05/2/1	85	6
- PTSA/Formic Acid/Turpentine	0.2/2/1; 0.05/2/1	85	6
- Sulphuric Acid/Formic Acid/Turpentine	0.2/2/1; 0.05/2/1	85	6

The α-pinene conversion (C_pinene_), terpineol selectivity (S_terpineol_) and the yield of terpineol (Y_terpineol_) were calculated according to the following equations:

(1) $$C_{pinene}=\frac{A_{pinene,feed}-A_{pinene,product}}{A_{pinene,feed}}\times 100\%$$

(2) $$S_{terpineol}=\frac{A_{terpineol}}{\sum A_{i}-A_{pinene,product}}\times 100\%$$

(3) $$Y_{terpineol}=C_{pinene}\times S_{terpineol}$$

where A_i_ and A_terpineol_ are respectively for the corrected chromatographic area of particular compound and terpineol in GCMS spectra. All experiments were conducted at a minimum of three repetitions to ensure the reproducibility of the data. The obtained average standard deviation errors of those three runs were below 8.2%.

## 3 Results and discussion

### 3.1 GC-MS spectra of product

**Figure 1:**
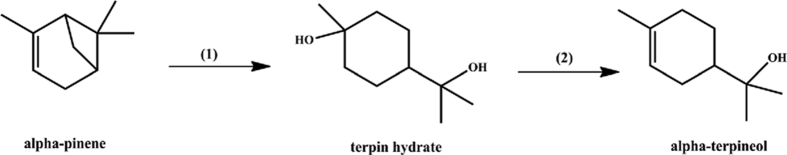
Reaction scheme of terpene conversion to terpineol.

Feed conversion, product selectivity, and yield values of the hydration reaction were obtained and calculated from standardized GC-MS spectra analysis. The example of spectra comparison between raw turpentine and oil-phase product after hydration reaction is shown in Figure 1.

**Figure 2:**
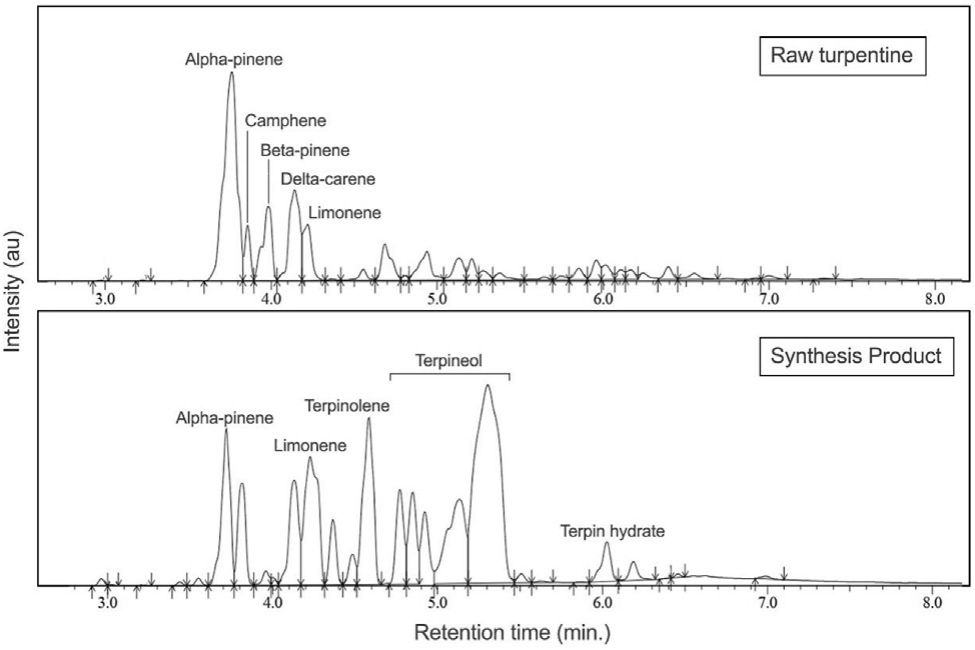
GC-MS spectra comparison of between feed (raw turpentine) and hydration reaction product (85 °C, 6 h, catalized by PTSA).

In Figure 1, it shows that in the feed (raw turpentine), no terpineol component was detected. Initial feed consists of α-pinene, camphene, β-pinene, δ-carene, and limonene. After hydration reaction, a smaller α-pinene and β-pinene peak was found and producing a higher concentration of camphene, δ-carene, and limonene (see Figure 2 bottom). Those components were defined as side products and competing for the formation of terpineol as the main desired product. Fortunately, those side products are usually more expensive than feed (pine oil) price in the market [2] but their small concentration in the product could create a major challenge for the purification. For the reaction mechanism itself, most plausibly the acid catalyst used in this experiment catalysed both hydration and dehydration consecutively. This phenomenon is proven by the formation of terpin hydrate in the reaction product. Despite its potential, PTSA as a strong acid catalyst tends to attack both hydroxyl groups within terpin hydrate molecule rapidly thus causing the formation of limonene and terpinolene. In addition to those composition (as mentioned above), the analysis also allows insight into the composition of the other structural components. Small amount of other mono-cyclic and bicyclic compounds was found in the feed which include p-cymene (1.2%), terpinolene (0.7%), β-myrcene (1.81%), and other unidentified aliphatic products (less than 3%). In addition, terpene alcohols such as menthol, terpinol, isoborneol, and esters, ketones and carboxylic acids have been identified. The identification of long chain terpenes was difficult due to the impossibility to identify the molecular ion, and some peaks were not identified accurately. As turpentine was the distillate product of liquid-solid gum rosin, slight amount of long chain terpenes could present in the turpentine due to equilibrium and soluble in the bulk liquid called as residue [24].

### 3.2 Optimum reaction condition of terpineol synthesis

**Figure 3:**
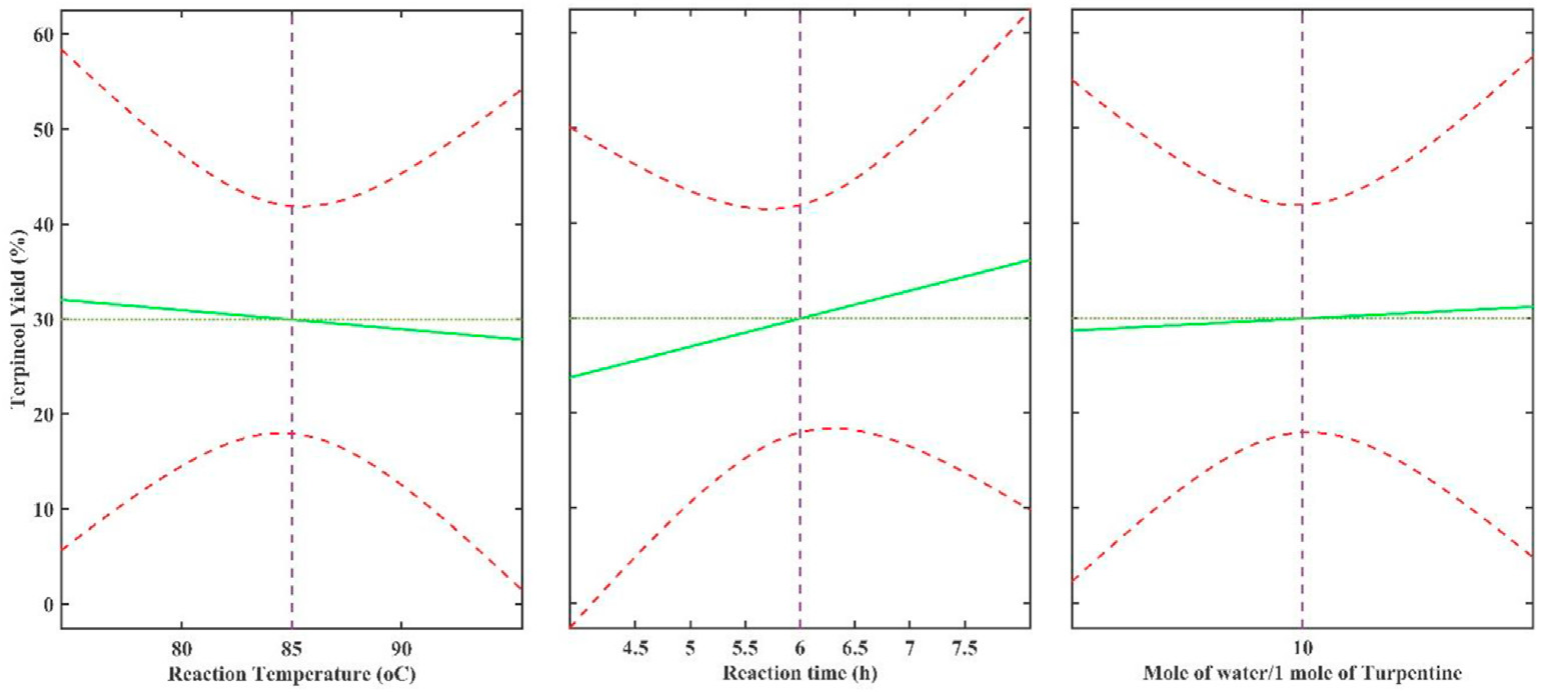
Results of response surface analysis generated by MATLAB® software.

In order to simply the optimization of catalysts type and composition preliminary experiment is conducted through systematic One Factor at A Time (OFAT) approach [25]. In this work some reaction parameters, *i.e*. reaction temperature, reaction time, and water to reactant ratio, are optimized by using PTSA as the selected catalyst following the result of previous work [14]. Thus, optimum reaction condition was determined based on the highest yield of terpineol using PTSA as catalyst and catalyst to reactant ratio of 1–10. PTSA was found to be the best catalyst for pure α-pinene hydration to terpineol according to our previous work [18]. The reaction was conducted three times to ensure the reproducibility of the data and the average result are presented in Table 2. The results from our previous work show that once the optimum condition was obtained for a certain strong acid-catalyst, the optimum condition for the following weak catalysts did not change significantly. Additionally, response surface analysis (RSA) of acquired data (yield vs reaction parameters) is conducted to ensure the optimum point acquired from above mentioned approach. From both OFAT approach and RSA, it can be observed that the optimum reaction condition is converged at 6h, 85 °C, and 10 mol/mole for reaction time, reaction temperature, and mole H_2_O/mole Turpentine respectively indicating that parameter types and selection from OFAT approach has adequately provided information for reaction parameters optimization (see Figure 3).

**Table 2:** Optimization of reaction parameters.

No.	Parameter	Value	Terpineol Yield (%-w/w)
1.	Reaction Temperature	75 °C	30.3
		85 °C	35.9
		95 °C	26.3
2.	Reaction Time	4 h	20.2
		6 h	35.9
		8 h	32.0
3.	Water amount (per 1 mol of turpentine)	5 mol	31.2
		10 mol	35.9
		15 mol	33.6

Following the result of Table 2, it can be observed that the optimum hydration temperature is 85 °C with terpineol yield of 35.9%. The optimum temperature is slightly higher compared to the optimum temperature of pure α-pinene hydration. A higher temperature is necessary to activate other more stable components in the raw turpentine. Compared to α-pinene, other monoterpene components, such as β-pinene, carene, and limonene, have higher stability and boiling point. However, at a higher temperature above 95 °C, an excessive amount of carbocations could be formed and resulted in a negative impact to the production of terpineol because the intermediate carbocation tends to isomerize rather than react with a water molecule.

Similar to the previous work [7], that conducted the experiment with a mixture of phosphoric acid and formic acid as catalysts, the optimum reaction time of this study was found around 6–8 h. Proper reaction time is required to avoid over-dehydration reaction that could result limonene which has almost similar reaction energy level compared to terpineol [14]. This result is also supported by previous work [8] that conducted the reaction in the range between 8 and 12 h. Although the hydrolysis of turpentine requires stoichiometry balance of 1 mol of water per 1 mol of reactant following the mechanism of pinene + H_2_O → terpineol, more water was required to ensure the reaction completion. Raw turpentine (classified as oil) and water were slightly soluble each other. Without the addition of emulsifier, such as Nonyl Phenol Ethoxylate (NP10), the reaction occurred only in the interphase layer between water and turpentine. Contrary, an excessive amount of water could lead to over-dehydration reaction into limonene. At pilot scale experiment, the best ratio of water to feed was found at 10–12 mol/mol. Hence, for further experiment, the operation condition was conducted at 85 °C, for 6–8 h, with water to feed ratio fixed at 10 mol/mol. In this work the usage of high temperature and prolonged reaction time need to be avoided since both aforementioned factors might lead to the dehydration of both hydroxyl group within terpin hydrate molecules. At this point, the preliminary experimental activities have fulfilled their purpose to select the most appropriate reaction condition so that the formation of both limonene and terpinolene as side products can be minimized.

### 3.3 Terpineol synthesis – variation of weak (organic) acid catalyst

**Figure 4:**
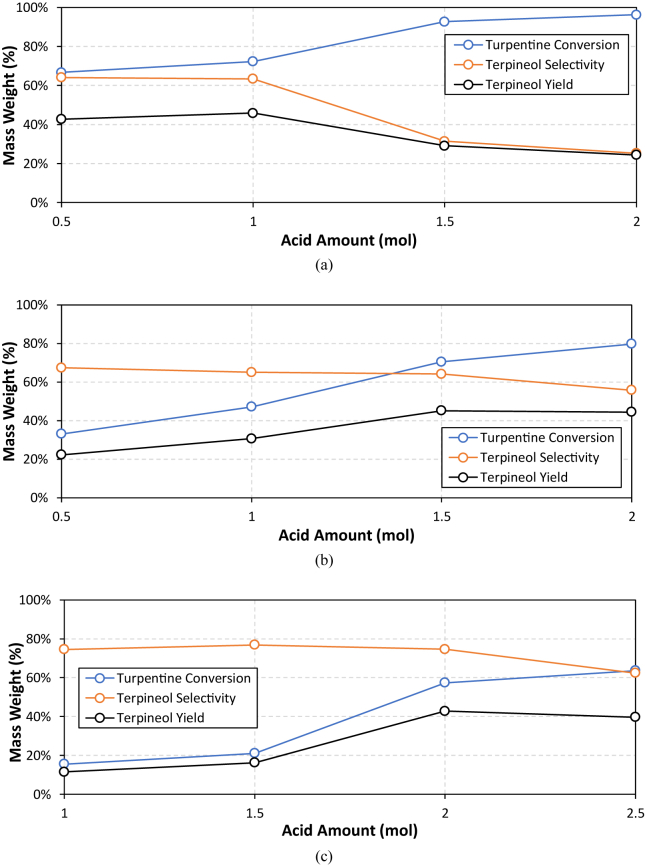
Influence of organic acid catalyst on terpineol production: (a) oxalic acid; (b) citric acid; (c) formic acid.

Single catalyst experiment was performed to determine the best ratio of weak/organic acid catalyst to feed in order to obtain the highest selectivity or yield of terpineol. Weak/organic acids used in this experiment are oxalic acid, citric acid, and formic acid. The experimental results for single catalyst variations are presented in Figure 4.

In general, the results show that turpentine conversion value tends to increase with the increasing amount of acid in the mixture but the terpineol selectivity trend was in the opposite condition. The increasing amount of acid will increase the concentration of intermediate carbocations hence the feed conversion is increasing. Once the carbocation was formed, the ratio between available water molecule in the system and intermediate carbocation will drive the reaction further. In the lower ratio, isomerization reaction will be dominant and result in a lower selectivity of terpineol. The highest yields for weak/organic acid catalyst experiment are 45.9% using oxalic acid (1 mol of oxalic/1 mol of turpentine), 45.2% using citric acid (1.5 mol of citric acid/1 mol of turpentine), and 42.8 using formic acid (2 mol of formic acid/1 mol of turpentine).

The physical properties of acids, such as acidity (pH) and catalyst solubility in the water (polar) and oil (nonpolar) phases, may affect the performance. Tables 3 and 4 show acidity values or dissociation constants (pKa) and solubility of acid catalysts in water (polar) and nonpolar compounds.

**Table 3:** Associated acid properties of each used catalyst and its optimum dosing.

No.	Catalyst	pKa	Maximum Yield of Terpineol	Optimum catalyst dosage (mole)
1.	Oxalic Acid	1.46	45.86 %	1
2.	Citric Acid	2.79	45.15 %	1.5
3.	Formic Acid	3.75	42.82 %	2

**Table 4:** Solubility values of organic acid catalysts.

No.	Catalyst	Solubility (g/L)	
		Water Phase	Nonpolar Phase
1.	Oxalic Acid	220 (at 25 °C)	Insoluble (Benzene)
2.	Citric Acid	592 (at 20 °C)	Insoluble (Benzene)
3.	Formic Acid	1000 (at 25 °C)	Slightly soluble (Benzene)

**Figure 5:**
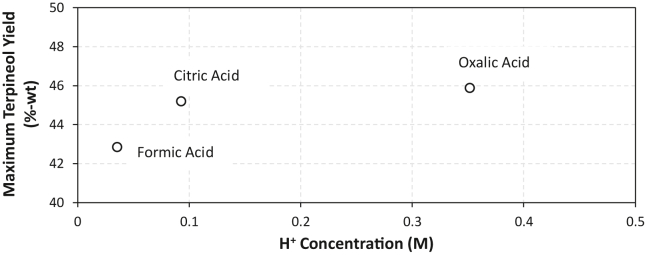
Effect of initial ion H^+^ concentration towards terpineol yield.

Based on Table 3, it can be observed that the trend of conversion follows the acid strength of the catalysts. The order from the highest and the lowest acid strength follows oxalic acid, citric acid, and formic acid. It is in accordance with the current experiment result that the highest to lowest turpentine conversion are following oxalic acid (67–97%) > citric acid (33–80%) > formic acid (16–64%). Following its conversion, after each catalyst initial proton concentration is being rationalized with each of its initial dosing (Table 3), a clear relationship between initial proton concentration and maximum terpineol yield can be drawn. As can be seen from the Figure 5, higher proton concentration leads to higher terpineol yield. This apparent results trend is in agreement with common concept of BrØnsted acid catalyst activity as a protonating agent [24].

Furthermore, in the case of solubility, all catalysts are generally soluble in the water (polar solvent), but some solubility differences were found in the organic solvent. Using benzene as the solvent, the solubility of those weak/organic acids follows this order: formic acid > oxalic acid and citric acid. The penetration of acid to the organic solvent is important to allow the catalyst to play a more major role in driving the reaction mechanism for terpineol formation. Following the experiments result, formic acid gave the highest terpineol selectivity of 62–77% and followed by oxalic acid and citric acid of 25–67%. High terpineol selectivity in formic acid clearly correlates to the ability of it to be dissolved in the organic phase. More acid catalyst in the oil phase layer could drive more water molecules to attack the carbocation of turpentine and form terpineol. In the case of oxalic acid and citric acid, these two acids are less soluble in the organic phase, therefore less influence on the hydration pathway. The intermediate carbocations tend to isomerize and form by-products such as terpinene and limonene. Our theoretical calculation showed that terpinene has the lowest reaction energy level compared to other derivatives of turpentine [14].

### 3.4 Terpineol synthesis - combined catalyst variation

**Figure 6:**
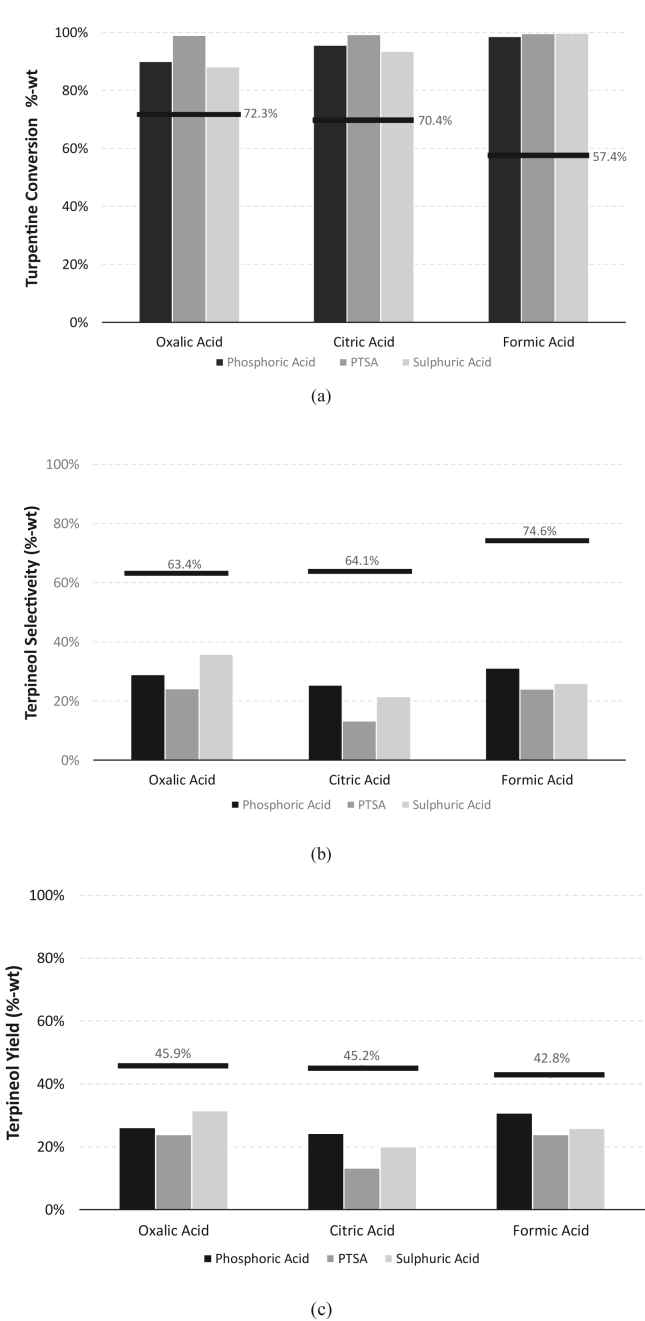
Influence of combined mixed acid catalyst on terpineol production at 0.2 mol strong acid/1 mol turpentine: (a) turpentine conversion; (b) terpineol selectivity; (c) terpineol yield.

The main purpose of strong/inorganic acids (phosphoric acid, p-toluenesulfonic acid (PTSA), and sulfuric acid) addition in the mixture is to increase the conversion of feed. In order to understand the phenomena, the experiment was conducted at two different values of acid ratios, high and low ratios. At high acid ratio, the strong acid to turpentine ratio was maintained at 0.2 mol/mol while at low acid concentration, the ratio was only 0.05 mol/mol. The experimental results for mixed catalyst variations at high acid ratio addition are presented in Figure 6.

**Figure 7:**
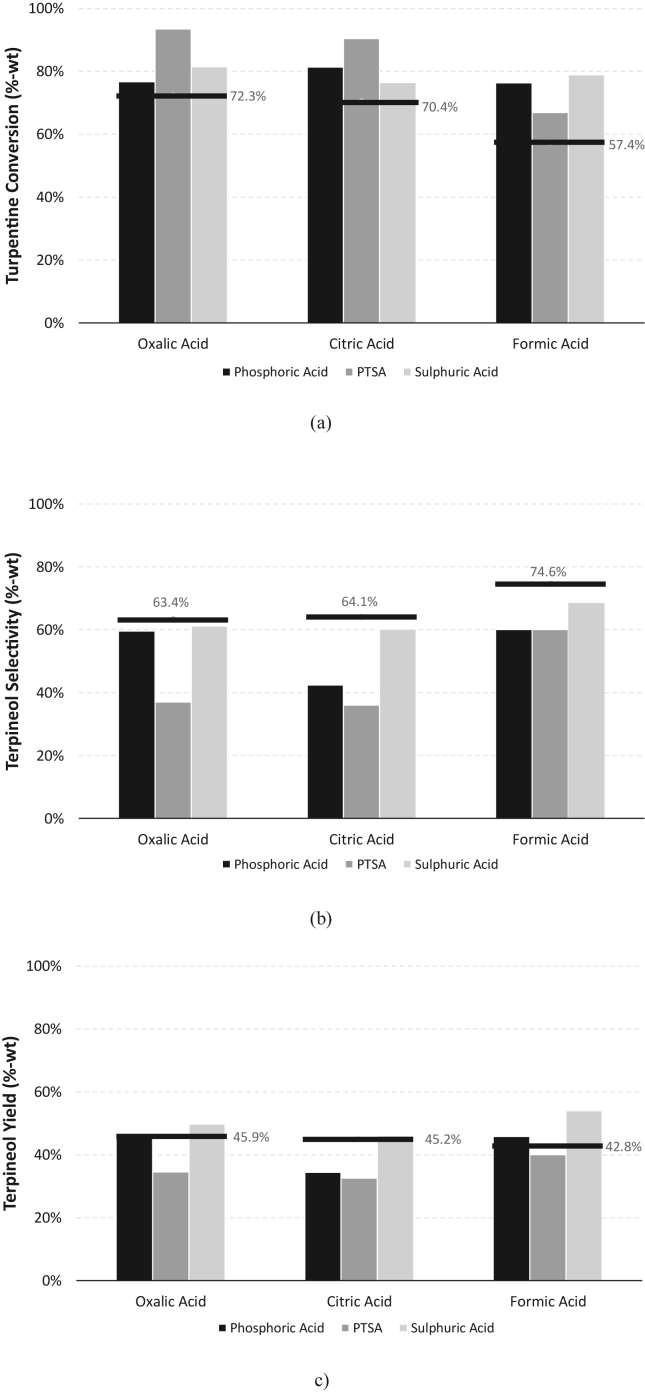
Influence of combined mixed acid catalyst on terpineol production at 0.05 mol strong acid/1 mol turpentine: (a) turpentine conversion; (b) terpineol selectivity; (c) terpineol yield.

Compared to the single weak/organic catalyst usage, the addition of strong acid catalyst could boost the conversion by 20–40% (see Figure 6, with single catalyst alone indicated by solid line). One apparent drawback is that terpineol selectivity decreased for about 30–50% compared to the usage of single catalyst and resulted in the final value of terpineol yield decreased by 10–20%. It can be concluded that the strong acid/inorganic acid addition (0.2 mol strong acid per 1 mol turpentine) did not increase the performance of terpineol production from turpentine. In the presence of higher concentration of strong acid, more intermediate carbocations was produced but weak/organic acid catalyst has less control in driving the reaction. This leads the reaction to be in random pathways. Lowering the strong acid ratio can possibly solve this problem and the result is shown in Figure 7.

Following the above result, it was observed that the lower ratio of strong acid to the feed increased the turpentine conversion moderately (increasing the conversion by 5–30% higher compared to single weak/organic acid). Terpineol selectivity also reduced but in a very small amount, about 2–15% for phosphoric acid and sulfuric acid and about 4–15% for PTSA. In total, final terpineol yield for the mixed catalyst at the low concentration of strong acid was higher than the terpineol yield of a single weak/organic acid. For example, the terpineol yield using single oxalic acid was 45.8% and increasing to 49.8% by the addition of sulfuric acid. The highest yield of terpineol was found at 54.2% when formic acid was combined by sulphuric acid. By comparing the data shown in Figures 4, 5, and 6 it can be inferred that there is a tendency for terpineol yield for either strong and weak acids. For weak acid case the amount of initial proton concentration plays important role to boost the yield of terpineol whilst for strong acid case the amount of initial proton atom originated from it need to be controlled so that uncontrolled dehydration reaction of terpin hydrate can be avoided. In this work, terpineol yield can be boosted by simply adding small amount of strong acid (see Figure 7).

## 4 Conclusions

This work consists of three main parts that can be mentioned as preliminary experimental (aimed to find and fix the reaction condition of future test), test of weak acid capability, and test of combined acid capabilities. For the result of preliminary experimental it was found that the most appropriate condition for further catalytic test is at 85 °C, 6 h, and 10 mol/mole for reaction temperature, length, and water to turpentine mole ratio respectively. Among weak acid catalyst used for catalysing terpineol production (single catalyst examination) Oxalic acid achieved the highest yield and this can be resulted from its highest initial proton concentration. For combined catalyst (weak and strong catalysts usage) formic acid combined with sulphuric acid at 0,05 mol/mole sulphuric acid condition gives the best terpineol yield at 54.2 ± 8.2%. The combination of diprotic strong acid and the weakest acid used in this work could lead to rapid terpene conversion without causing excessive production of limonene and terpinolene as the product of non-selective dehydration reaction of terpin hydrate. Despite the observed tendency of initial proton concentration as the one that affects the terpineol yield significantly future research activities should be able to conduct an examination whether available proton concentration is the only significant factor or there is another factor such as solubility of acid and other factors. In term of increasing terpineol yield, future research should be able to utilize the combination of both organic acid (soluble in turpentine oil) and acid heterogeneous catalysts so that both solubility and non-selective dehydration issues can be solved.

## Declarations

### Author contribution statement

Tirto Prakoso & Lienda Handojo: Analyzed and interpreted the data.

Ilham Ardiyanto Putra: Performed the experiments; Wrote the paper.

Tatang Hernas Soerawidjaja: Conceived and designed the experiments.

Haryo Pandu Winoto: Contributed reagents, materials, analysis tools or data; Wrote the paper.

Antonius Indarto: Conceived and designed the experiments; Analyzed and interpreted the data; Wrote the paper.

### Funding statement

This work was supported by the 2020 10.13039/501100003693KIST School Partnership Project between the Korea Institute of Science and Technology (KIST) and the Institut Teknologi Bandung (ITB), and 10.13039/501100015689was partially funded by the Indonesian Ministry of Research Technology/National Agency for Research and Innovation, and the Indonesian Ministry of Education and Culture under the World Class University (WCU) Program managed by Institut Teknologi Bandung. The author would also like to thank Perhutani Pine Chemical Industry (PPCI), Pemalang, Indonesia for the research support.

### Competing interest statement

The authors declare no conflict of interest.

### Additional information

No additional information is available for this paper.
