# Supplementary material for: A method to control terpineol production from turpentine by acid catalysts mixing
Source: Heliyon. 2020 Oct 8;6(10):e04984. doi: 10.1016/j.heliyon.2020.e04984 (PMC7550928; doi:10.1016/j.heliyon.2020.e04984)
Supplement: Supplementary Material-Figure S1 [file mmc1.docx]

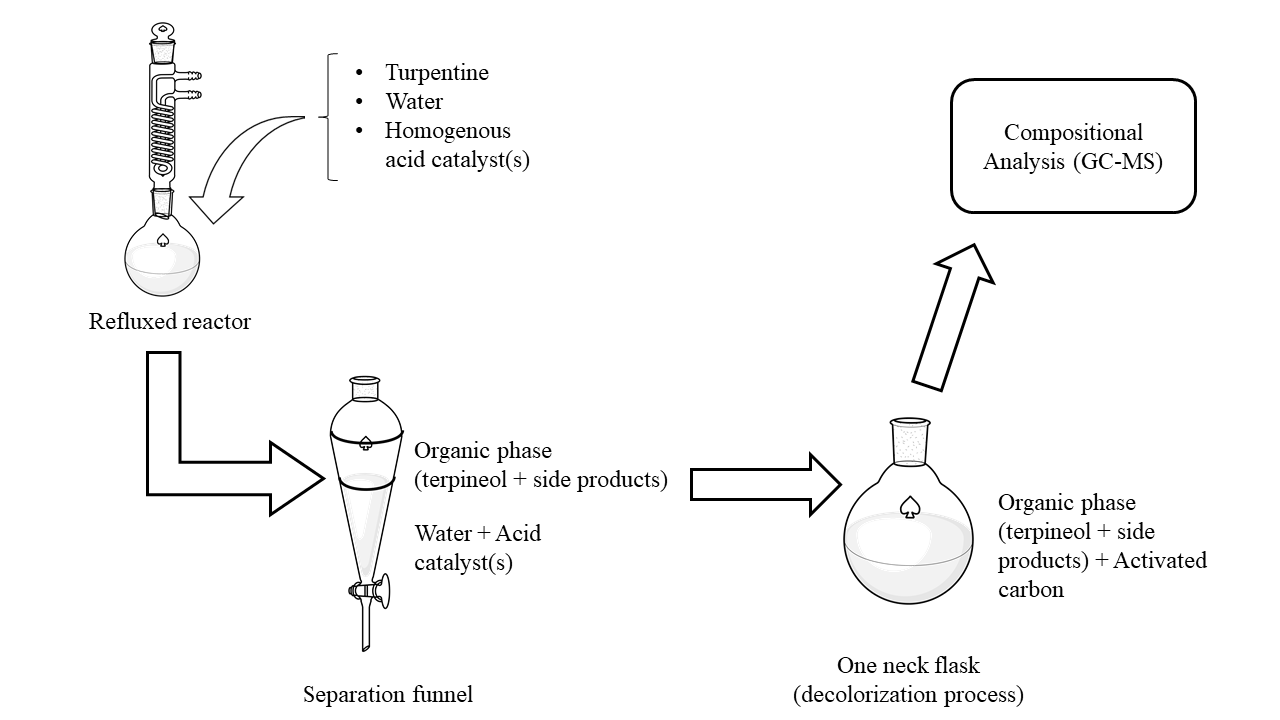


Figure S1. Experimental procedure for one-pot catalytic reaction of terpineol production from turpentine
