# Supplementary material for: A method to control terpineol production from turpentine by acid catalysts mixing
Source: Heliyon. 2020 Oct 8;6(10):e04984. doi: 10.1016/j.heliyon.2020.e04984 (PMC7550928; doi:10.1016/j.heliyon.2020.e04984)
Supplement: Supplementary Material-Figure S2 [file mmc2.docx]

(a)

(b)

Figure S2. Calibration GC Curve for Pinene and Terpineol
